# Using interpersonal communication strategies to encourage science conversations on social media

**DOI:** 10.1371/journal.pone.0241972

**Published:** 2020-11-10

**Authors:** Curtis Martin, Bertrum H. MacDonald

**Affiliations:** 1 Ocean Frontier Institute, Dalhousie University, Halifax, Nova Scotia, Canada; 2 School of Information Management, Dalhousie University, Halifax, Nova Scotia, Canada; King Abdulaziz University, SAUDI ARABIA

## Abstract

Today, many science communicators are using social media to share scientific information with citizens, but, as research has shown, fostering conversational exchanges remains a challenge. This largely qualitative study investigated the communication strategies applied by individual scientists and environmental non-governmental organizations on Twitter and Instagram to determine whether particular social media practices encourage two-way conversations between science communicators and citizens. Data from Twitter and Instagram posts, interviews with the communicators, and a survey of audience members were triangulated to identify emergent communication strategies and the resulting engagement; provide insight into why particular practices are employed by communicators; and explain why audiences choose to participate in social media conversations with communicators. The results demonstrate that the application of interpersonal communication strategies encourage conversational engagement, in terms of the number of comments and unique individuals involved in conversations. In particular, using selfies (images and videos), non-scientific content, first person pronoun-rich captions, and responding to comments result in the formation of communicator-audience relationships, encouraging two-way conversations on social media. Furthermore, the results indicate that Instagram more readily supports the implementation of interpersonal communication strategies than Twitter, making Instagram the preferred platform for promoting conversational exchanges. These findings can be applicable to diverse communicators, subjects, audiences, and environments (online and offline) in initiatives to promote awareness and understanding of science.

## Introduction

Human activities—both past and present—are having detrimental impacts on the earth’s environmental systems: fishing practices have forced fish stocks to critical condition [1], many of the planet’s species are being driven to extinction at an alarming rate [2], and continuous burning of fossil-fuels has created a global climate emergency [3]. If these harmful environmental practices are to be mitigated, they need to be managed through policy decisions at the science-policy interface where various actors, barriers, and enablers affect the flow of information from researchers to decision-makers [4]. Citizens are an important group that interacts with numerous stakeholders at this interface. If citizens are to be effective participants in decisions and solutions to address deteriorating environmental conditions, relevant research information must be communicated effectively to this diverse group. However, this communication is not a trivial activity, as cultivating environmental science literacy has proven to be a major challenge [5–8]. Climate change literacy is often cited to illustrate this challenge; misunderstanding is still widespread among citizens, due to a combination of denial, intentional obfuscation of facts, and personal values taking precedent over scientific information [5, 6, 9, 10].

Although risks are associated with communicating science via social media (such as being subject to internet trolls and anti-science users [e.g., 11, 12]), the internet and social media provide science communicators with significant opportunities to share policy-relevant information with citizens, as such tools are now the main information source for the public, including for scientific and policy information [13, 14]. As of 2019 an estimated 4.4 billion people use the internet, with nearly 3.5 billion active on social media [15]. The latest statistics show that billions of social media posts are created daily on Facebook, Twitter, Instagram, YouTube, and other social media platforms, and the numbers are increasing [15, 16]. Although important barriers to internet access still exist [e.g., 17, 18], new media are generally user-friendly and widely available; simple and quick web searches can break down technical and financial barriers to information, and social media platforms are primarily inexpensive and accessible internationally [19, 20]. Virtual communities can be formed online to facilitate public engagement with science, and citizens now have greater opportunity to participate in science communication, bypassing traditional information “gatekeepers” (e.g., scientific journals, popular media, government reports) to aid in information dissemination, and increase public awareness of important scientific issues [19, 21–23].

Numerous researchers have explored whether relationships exist between social media posting behaviours of communicators and audience engagement [e.g., 24–30]. Research on this subject has been mainly exploratory to date, with studies covering a range of social media platforms and methods. At present, the results indicate that communication techniques can play an important role in generating audience engagement for both individual and organization communicators, but that science communicators have typically struggled to encourage conversations on social media, particularly with citizens exposed to such information for the first time [31–33]. Some studies have noted that science communicators have given lower priority to strategies that would promote engagement via online conversations [34]. Researchers have called for further exploration to understand better the challenges of facilitating science conversations on social media, to identify additional means of improving engagement, and to investigate whether communicator strategy and audience engagement patterns persist across communication topics [25, 29, 30, 35]. In particular, they have called for small scale studies that offer detailed insights that big data approaches are less likely to provide [35].

This study applied a mixed methods approach to investigate communication strategies and two-way conversation activities of individual and non-governmental organization science communicators on two different social media platforms (Twitter and Instagram). The study triangulated data obtained through qualitative methods to: identify emergent communication strategies and resulting audience engagement; gain insight into why particular practices are employed by communicators; and determine why members of the audiences choose to participate in social media conversations with communicators.

## Literature review

### Science communication on social media

The ability to communicate science to a wide variety of audiences is important. Scientific information is often needed for effective policy decisions, and strong science communication can promote the use of relevant information in environmental decisions [4, 36, 37]. Scientific information should be actively shared with citizens. Not only is the majority of scientific research publicly funded, citizens also need access to scientific information to make informed input to decisions on subjects relating to public policy, technological advancement, political preferences, and personal environmental practices, among others [26, 38–42]. Communicating science to audiences beyond the academic community is increasingly seen as a responsibility of scientists, and is in some cases central to receiving research funding [40, 43–45].

Scientists have been turning to social media to communicate the results of their research [46, 47]. These media are significant because they grant communicators a platform for two-way exchanges with members of the public. Previously, the common and accepted communication model was based on resolving a perceived knowledge deficit to improve public understanding of science [48–50]. In this “first-order” way of thinking it was assumed that citizens lacked knowledge and acted as passive receivers of information. Thus, solely providing people with the necessary information was intended to lead to greater understanding and awareness of public issues [48, 49, 51, 52]. “Second-order” communication that is reflexive, deliberative, and depends on dialogic, two-way information exchange is now thought to be a better model for sharing information with citizens [49, 51, 52]. This latter model promotes knowledge co-production between researchers and citizens by allowing people to bring their ideas and values to the conversation, and facilitates the formation of trust relationships between researchers and citizens [48, 49, 53–56]. A third participation model of science communication has also been proposed in the belief that all involved can contribute to decisions that affect them [57, 58]. Social media—including blogs, microblogs, social networks, podcasts, and curatorial tools—offer the potential to facilitate deliberative communications, allowing citizens to participate in research discussions online by responding to information, sharing it with others, and/or creating new science communication resources [46, 59, 60].

### Non-governmental organizations and individual scientists as communicators on social media

Social media have become significant to organizational practice [61–63]. Non-governmental organizations (NGOs) in particular have been credited with pioneering the use of social networking tools, prior to their use by government agencies and private companies [64]. As a result, social media—including Twitter and Instagram—are used by many NGOs around the world. According to a recent report, 77% of NGOs use Twitter, and 50% use Instagram, with the majority posting on both Twitter and Instagram at least once per week [64]. NGOs of all sizes are reaching large numbers on both platforms with some building massive audiences. For example, Amnesty International has over 1 million Twitter followers (www.twitter.com/amnesty), and over 500,000 Instagram followers (www.instagram.com/amnesty).

NGOs cite numerous benefits associated with social media use, including fundraising, increased brand awareness, volunteer recruitment, improved event organization, and more effective communications [64–66]. Through social media, organizations can share information, participate in conversations, and build relationships with their audiences [65–68]. Nonetheless, various studies show that NGOs have not fully capitalized on the affordances granted by social media: organizations have typically been found to focus on one-way communication models characteristic of a knowledge-deficit, using social media primarily as a broadcast tool, similar to the practices observed for some government agencies [25, 29, 68–72].

Individual scientists have been relatively slow in adopting social media [73–77]. According to a survey by Nature, an estimated 13% of scientists use Twitter regularly, with 50% of those engaging in scientific discussions on the platform [78]. According to another study, it is estimated that a smaller portion of scientists active on Twitter also use Instagram [79]. One reason for slow acceptance is that science outreach is often not incentivized for researchers; researchers interested in communication activities are therefore often required to pursue them on a volunteer basis in addition to their professional duties, creating a time barrier [79, 80]. Furthermore, scientists—especially those working in government and industry—are sometimes discouraged from open communications [e.g., 81–83]. In other words, broad and public communication is typically not regarded as a valuable activity for researchers [79]. There is also evidence that individual scientists avoid communicating via the tools due to a general lack of knowledge on how the tools function, questions surrounding the rigor of scientific discussions on social media, and incorrect perceptions that the tools are ineffective as a means of scientific communication [75–77, 79].

Numerous studies have demonstrated the strong communication potential that social media provide to scientists [e.g., 84–86]. Social media afford scientists the ability to build their “personal brand” by communicating their research and other related subjects [86]. Additionally, social media provide an avenue through which scientists can communicate to the public, which, although not new, is a more common and more requested pursuit for researchers today [87–90]. However, research shows that scientists utilizing social media are mainly sharing research within their own fields, with outreach to the wider public remaining a lower priority [75–77, 79]. Some scientists also over-emphasize the importance of blogs as a tool for communicating with public audiences; blogs were previously thought to be useful for encouraging dialogues with citizens, but in practice have not been widely successful in reaching non-scientific audiences [79, 91].

As illustrated above, science communicators have had difficulty in engaging citizens in two-way conversations on social media, which has led to calls for more innovative/inventive strategies to engage citizens with research, predominantly on subjects linked to important public policy issues [e.g., 92]. Furthermore, social media communication strategies often vary among communicators, including individuals and organizations, which affect whether communication is effective [e.g., 69, 93].

This study investigated strategies to engage people with scientific and policy information on social media. Research indicates that social media practices can affect how audience members engage with posts shared by individual and organization communicators [31]. Therefore, the first research question addressed by this study is:

RQ1: How do individual and NGO communicators approach sharing scientific and policy information on social media, and what particular strategies do they apply in their activity to engage with their audiences?

Furthermore, science communicators have typically struggled to encourage conversations on social media, despite evidence of two-way conversations being more effective for information sharing than one-way transmission [32, 33, 49, 51, 52]. Therefore, the second research question addressed by this study is:

RQ2: Do particular social media strategies encourage two-way conversations between science communicators and online audiences, and what characteristics of the strategies encourage communicators and audiences to participate in two-way conversations?

The goal of this research was to identify communication practices that encourage two-way conversations between communicators and citizens on social media. If particular techniques are more engaging, they could be adopted or prioritized by communicators to improve how scientific and policy information is shared on social media, and ultimately enable citizens to participate in decision-making processes.

## Methods

**Fig 1:**
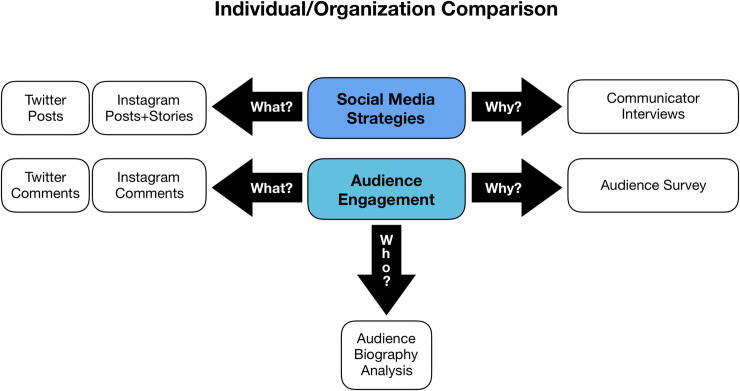
Research framework outlining the study design/methodology.

To address the research questions, the activity of four scientists acting as recognized science communicators using individual Twitter and Instagram accounts and the activity of three environmental non-governmental organizations (eNGOs) using organization Twitter and Instagram accounts to share scientific and policy information were studied. This number of communicators was selected to consider the research questions in a detailed, qualitatively data-rich manner (consistent with calls for such studies; [e.g., 24]) rather than be representative of all scientists and eNGOs communicating on social media. This study was conducted with established qualitative research methods appropriate for the sample size of communicators and volume and types of data collected [e.g., 94]. This research included: 1) an analysis of public Twitter and Instagram data of each of the seven account holders to identify practices implemented by communicators and resulting follower engagement in two-way conversations; 2) interviews with the individual and eNGO communicators to determine their social media strategies; 3) a survey of audience members involved in two-way conversations to determine why they participate in conversations on social media; and 4) an audience “biography” analysis to determine whether the communicators are engaging a scientific, non-scientific, or mixed audience on social media (Fig 1). Following collection, the aggregated social media data were triangulated to develop thorough understanding of social media strategies used by the communicators.

Ethics approval for this study was obtained in the ethics review process established by the Social Sciences and Humanities Research Ethics Board at Dalhousie University. As required by the ethics approval, informed consent was obtained from the participants prior to the interviews and the survey. The social media data collection complied with the Terms of Service for both Twitter and Instagram. Twitter was selected for this study because it is actively used for science communication and has been studied to a greater extent than other platforms [35, 75–78]. Instagram was selected because it is a newer platform, and fewer studies on the potential of Instagram as a science communication tool have been completed to date [35, 78]. Studying usage of the two platforms, which offer different features, allowed determining whether the communicators were consistent in their application of social media strategies.

### Account identification

Following the requirements of ethics approval, all of the participants were treated anonymously. The four individual scientists are located in four countries in North America and Europe. These scientists were chosen from The SciCommunity, an Instagram community that uses social media to make science, technology, engineering, arts, and mathematics more accessible (instagram.com/thescicommunity). The individual communicators were selected based on the order in which they joined the community. Beginning with the earliest community members (i.e., most established communicators), scientists who use personal Twitter and Instagram accounts to communicate primarily in English frequently each week, with accumulated 10,000 followers or more (Twitter and Instagram combined), were invited to participate in the study. Invitations were extended until four communicators agreed to participate in the study. The three eNGOs, also with many thousands of followers, were selected for their focus on sharing environmental information on Twitter and Instagram regularly each week, and for the scale of the organization (one local, one national, and one international). Invitations were extended to eNGOs that met the criteria until three agreed to participate in the study, Environmental NGOs were studied due to their growing role as science communicators to diverse audiences [63, 95].

### Social media data collection and coding

Publicly available Twitter and Instagram data posted by the seven communicators were collected for four weeks from July 30 to August 26, 2018, including all Twitter posts (TRPs), Instagram posts (IGPs), Instagram stories (IGSs), and all associated TRP and IGP comments. As this study followed a largely qualitative approach to investigate the social media practices of the communicators, one month was judged to be sufficient for analysis and triangulation with the interviews. During the interviews (see below), communicators were asked to focus their responses on their most recent social media activity. Twitter posts were collected once per day using the desktop version (twitter.com) one week after they were posted to allow time for audience engagement (from August 6 to September 2, 2018). A screenshot of the TRPs recorded the date/time of posting, captured images, and preserved a “snapshot” of the content and engagement. In the case of multiple Twitter posts together (i.e., a thread), the posts within a thread were captured and treated as a single post, unless posts occurred over multiple days.

Instagram posts were collected from the desktop version (instagram.com) in the same manner as TRPs. Instagram stories were collected twice daily to ensure none were missed (as stories expire after 24 hours). Screen capture software was used to record the video and audio associated with each IGS post. Each set of stories was saved as a video file and the stories were separated into threads based on the time between posting and topic continuity. Engagement data from IGSs are not public and were not captured.

The Twitter and Instagram data were organized in spreadsheets for statistical analysis in Rstudio version 1.1.456. For the TRPs, five spreadsheet files were created: original content, comments, handles, names, and reply type (response from the original communicator vs. a secondary social media user). The content files contained two columns—post caption data, and hashtag data—with each row representing a unique post. The other files were organized similarly, with each row containing data on either comments, handles, names, or reply types associated with a unique post. This process was used for IGPs, but only for original content, comments, and handles were created, as data for names and reply type are not recorded within Instagram posts. Each TRP, IGP, and IGS was categorized for the content characteristics [S1 Table] using codes based on topics listed as central to the goals of organizations, and the Instagram description for The SciCommunity. Because the Instagram story data were recorded in audio/visual formats, rather than text, the IGSs were only subjected to content coding. In total, 840 social media posts and 1399 comments were collected and analyzed.

## Text analysis

The Twitter and Instagram post captions were subjected to text analysis using Linguistic Inquiry and Word Count 2015 (LIWC2015) software, which was used to identify the percentage of personal pronouns used in social media posts by the communicators, as such pronouns can affect how interactions between communicators and their audiences are perceived [96]. LIWC has been validated and used in numerous published research studies [75, 97]. English and non-special character data in the text captions posted by each communicator were analyzed as a single dataset, aided by Excel. The analysis was conducted separately for the Twitter and Instagram data for each communicator. Individual and eNGO scores were aggregated, as both communicator groups were analyzed under the same conditions.

### Interview data collection and analysis

The owners or representatives of the seven accounts were invited via email to participate in semi-structured interviews and to maintain anonymity were randomly assigned a code (e.g., IND1 for an individual scientist or ORG1 for an eNGO). The interview questions were designed to investigate how the communicators viewed their use of social media generally, along with their goals/objectives, their posting strategies, and their participation in social media conversations. The interviews, conducted by phone or Skype, were audio recorded. The interviews were transcribed verbatim and subjected to three rounds of coding, following established analysis processes [98–100], to draw out the themes from the textual data: an initial round to determine specific codes for each relevant interview response, a second round to create broader grouping of associated codes into categories, and a final round to restructure categories into overarching themes of all interviews. In the initial round, coding was conducted by one researcher, followed by a second researcher. The coding was compared and where discrepancies occurred, the researchers discussed the variations and resolved the differences. In subsequent rounds as the themes were drawn from the underlying coding, the second researcher reviewed the theme extraction to ensure consistency of application.

### Survey data collection and analysis

An online survey, open from September 10 to October 31, 2018, was administered using Opinio software to query engaged users about their participation in two-way social media conversations. Individuals who posted English comments in two-way conversations on Twitter or Instagram posts of each of the accounts were invited to complete the survey. The participants were invited if they were involved in a conversation with a) one of the communicators in the study, or b) another user who commented on a communicator post. A two-way conversation was defined as a comment that received at least one response, with both the commenter and respondent invited to complete the survey. Accounts that were deleted or changed to a different “handle” by users before invitations were sent out, accounts that did not belong to individuals, accounts that were obvious trolls/bots (based on their social media profile and/or comments), and the seven accounts of the individual scientists and eNGOs in the study were excluded. A total of 425 conversationalists were invited to participate in the survey via either Twitter or Instagram (i.e., the platform in which a conversation occurred) using a unique comment that tagged the individual in a Twitter or Instagram post and asked to follow a link that directed them to a webpage containing the survey link. When users conducted conversations on posts of more than one of the accounts in the study, random selection was used to decide which account the user was contacted about. The participants were treated anonymously and limited to completing the survey once. The quantitative data were subjected to descriptive statistical analysis, and the free text responses were coded for content themes.

### Audience analysis

The Twitter and Instagram biographies of the individuals invited to complete the survey were analyzed statistically with the aid of Rstudio version 1.1.456 to determine if they self-identified as scientists on social media. The individuals were classified as scientists if their biography mentioned science or science disciplines (e.g., neuroscientist, biochemistry), or if their social media profile pictures clearly depicted them as scientists.

## Results

Because the aim of this study was to investigate the relationship between communication techniques and audience engagement, particularly two-way conversations across Twitter and Instagram, analysis of the activity data from the two social media platforms, the interviews, and the survey text responses and demographic information were integrated for each communicator in the presentation of the results. This approach triangulates each communicator’s social media practices (both their views about their strategies and actual practices) with audience engagement, while highlighting similarities and differences in the strategies and engagement between each communicator and as either an individual communicator or eNGO. Because this study connects the application of strategies and resulting engagement throughout the social media activity of the communicators, social media data were analyzed in aggregate (i.e., strategies and engagement across all posts), rather than on a post-by-post basis.

### Three strategy filters

The interviews and the Twitter and Instagram data show that the two communicator groups utilize three types of “filters” to guide their posting activity. First, the seven communicators operate within implicitly accepted social practices on each platform (i.e., platform conventions). Second, the two communicator groups apply specific activity strategies related to posting frequency and type of media used in posts. Third, the seven communicators implement interpersonal communication strategies in their posts. These three filters are implemented in a hierarchical manner, that is, the activity strategies are applied according to platform conventions, and the interpersonal strategies are applied in accordance with both the activity strategies and platform conventions. Interpersonal communication and strategies emerged as important characteristics of the communicators’ social media activity. Interpersonal communication has been the focus of extensive research [101–104]. The succinct definition by Braithwaite, Schrodt, & Carr [105], “interpersonal communication is the production and processing of verbal and nonverbal messages between two or a few persons,” is pertinent in this study as this definition accounts for communication centred on individuals, focused on interactions involving exchange of messages, and on development of relationships between the participants. As is shown below, the strategies that communicators implemented to promote interpersonal communication gave attention to one or more of these aspects.

### Platform conventions

The interviews with the seven communicators show that accepted social media conventions play an important role in dictating the techniques applied by them, as they recognize that adherence to the common platform practices that have emerged over time will ensure their posts remain consistent with the expectations of social media users. The communicators expressed similar views of how they plan and implement strategies based on the platform conventions. For example, all of the communicators noted that Twitter tends to attract a more educated and/or issue-cognizant audience seeking news-centric information, and that Instagram draws a larger general/non-scientific audience interested in more personal multimedia posts, and therefore the seven communicators post accordingly to meet audience expectations (e.g., “You can share photos on Twitter, but it’s more visible and accessible on Instagram” (IND 4 interview)). Additional strategies applied by the communicators (discussed below), are implemented in compliance with implicit platform conventions.

### Activity strategies

**Fig 2:**
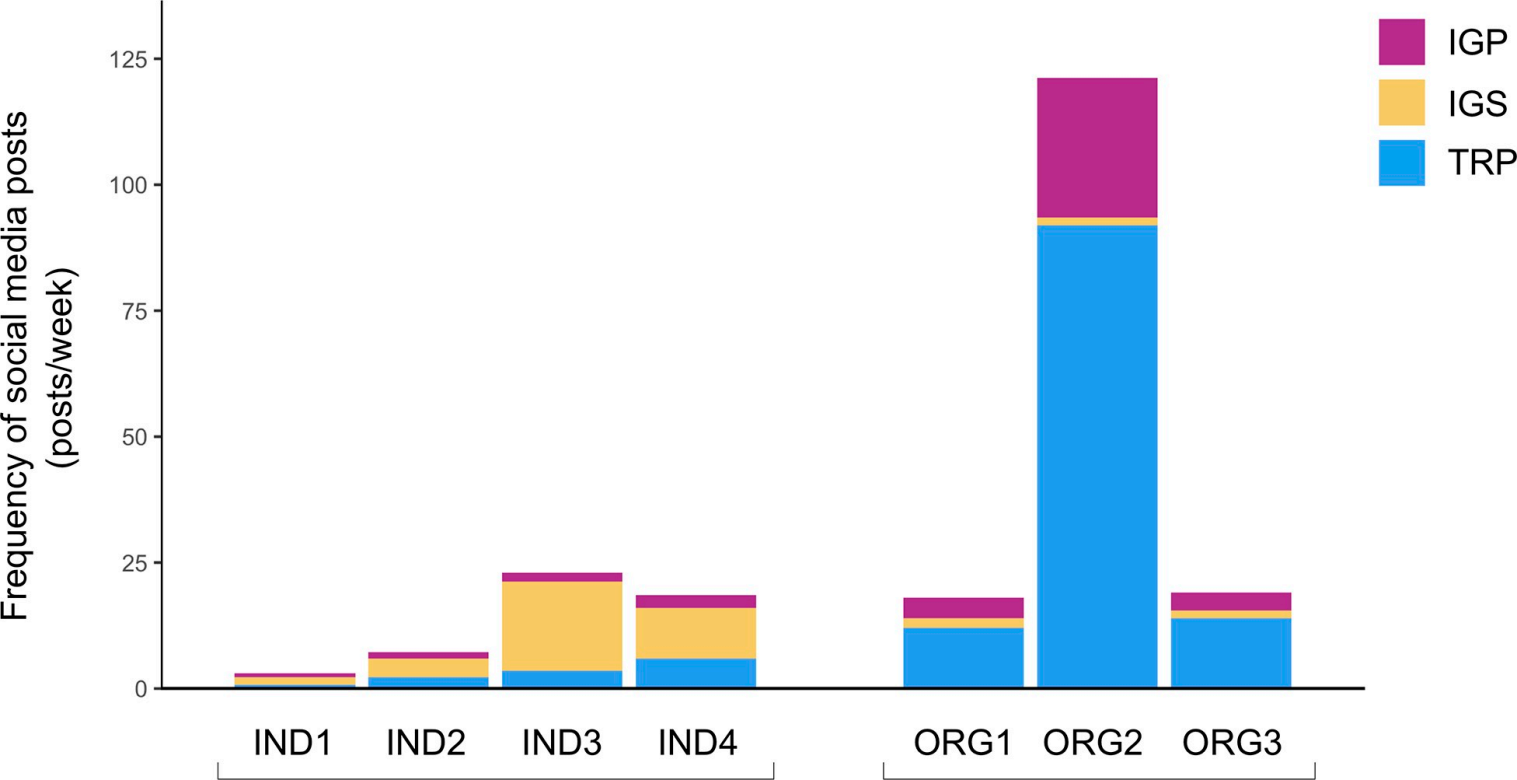
Average number of social media posts per week by individuals (IND) and eNGOs (ORG), July 30-August 26, 2018. Colours indicate the platform distribution of Twitter posts (TRPs), Instagram stories (IGSs), and Instagram posts (IGPs).

The individual and eNGO communicators implement particular strategies related to post frequency, platform priority, and media type used in posts—hereafter referred to as activity strategies—although with some variability. The eNGOs strive to post at regularly scheduled intervals, while maintaining flexibility to react when necessary. For example, one eNGO representative stated: “[we’re] doing as much planning as possible, but trying to leave in the flexibility to react when there is a more timely or necessary content need” (ORG2 interview). This approach allows the eNGOs to present well-researched information that is backed by evidence, while still giving the organizations an opportunity to share topical content and participate in social media “conversations” regarding breaking news or unexpected events related to their work (e.g., an interesting animal encounter during field work). In practice, ORG1 and ORG3 post on social media about 20 times/week (Fig 2). ORG2, however, posts on Twitter and Instagram much more frequently, at a rate of >120 times/week (Fig 2), because it “seems to be the most effective” for encouraging engagement (ORG2 interview). The individual scientists post in a less scheduled manner than the eNGOs, mainly when they feel inspired to do so. IND3 and IND4 post at similar rates to ORG1 and ORG3 (about 20–25 times/week), but IND1 and IND2 less than 10 times/week (Fig 2). The individual scientists indicated that frequency is not as important as quality. They typically share based on more mentally “dynamic” factors (e.g., creativity, curiosity, inspiration, interest), and consequently do not feel motivated to post at high frequencies, which the individuals find to be overexerting or time consuming. As one communicator said, “I’ve kind of come to the point where it’s best for me just to post when I like, when [it] suits me best” (IND4 interview). Although the individual scientists did not discuss whether posting at high frequencies is an effective engagement strategy (other than ensuring the time between posts is not excessive, e.g., weeks), they did mention that they believed that the excitement/passion they are able to convey based on inspiration can be quite engaging for their audience.

The communicators decide which platform they use based on a mix of platform affordances and level of engagement received. However, the eNGOs and individual scientists do not prioritize the same platforms, in regard to intended strategies, or how they are translated into practice. ORG2 prefers Instagram over Twitter, as Instagram is seen as more aligned with the organization’s overall goals: “our preference, or our top performing platform I should say, has been Instagram … it’s still at a point of very rapid growth and evolution in terms of the functions or things you can and can’t do on the particular platform. So that’s lent itself to being a top performer” (ORG2 interview). ORG1 and ORG3 do not have expressed platform preferences. Nevertheless, based on actual post frequency, all three of the eNGOs prioritize Twitter over Instagram, sharing most of their posts (67–76%) on Twitter (Fig 2). For ORG2, this practice is not consistent with the stated platform preference noted during the interview. All three of the individual scientists said they prefer Instagram—especially IGSs. For example, IND3 emphasizes posting on Instagram because that is “where [my] biggest audience is,” while also noting the importance of functionality: “I love how many dimensions there are to using Instagram. You can do pictures, you can do posts, you can do videos and stories, you can live stream. It’s so … versatile in how you can use it that it’s been incredible as a creator” (IND3 interview). The actual post frequency corroborates the interview responses of the individual scientists, as 69–85% of all their social media posts were shared on Instagram, particularly IGSs, with 50–77% of all posts shared via IGSs (Fig 2).

**Fig 3:**
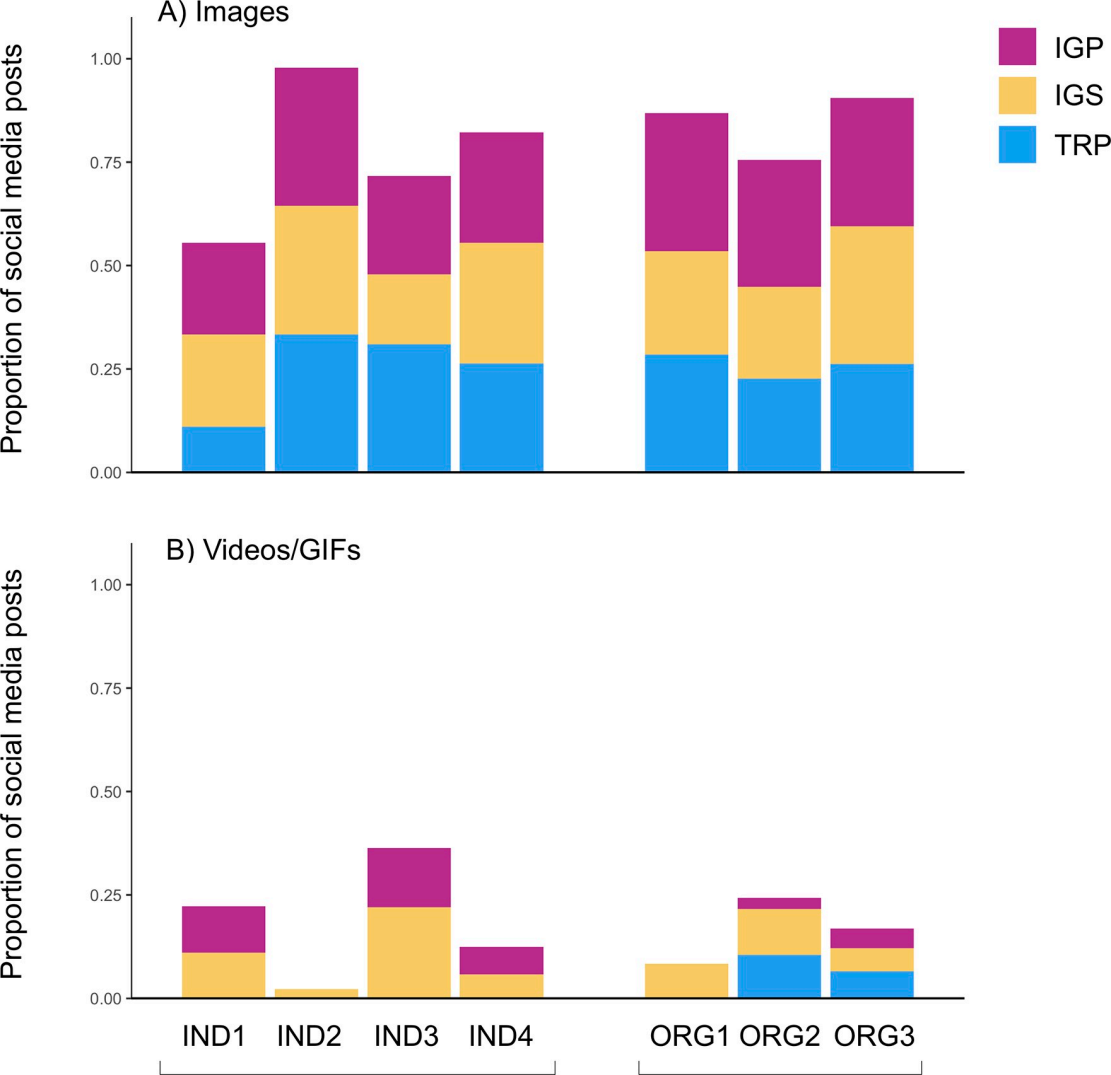
Proportion of social media posts by individuals and eNGOs containing A) images, and B) videos/GIFs, July 30-August 26, 2018. Colours indicate the relative proportion of posts with images or videos/GIFs in the TRPs, IGSs, and IGPs.

All of the communicators post text, images, and videos in accordance with platform conventions. The two groups of communicators use media types (text, images, and video) in a similar proportion of posts, but the individuals use text differently. Both the individuals and the eNGOs include text in all posts, images in the majority of posts (56–98%), and videos in a smaller fraction of posts (2–36%) (Fig 3). However, on Instagram, where the character limit is 2200 for each post, the individuals post an average of 244 words/caption, whereas the eNGOs use fewer words (an average of 102 words/caption) (Table 1). On Twitter, where the post length is more limited (280 characters), all communicators post a similar average of words/caption (28 for eNGOs and 30 for individuals) (Table 1). In addition, none of the individual scientists use Twitter to share videos, whereas two of the three eNGOs do (Fig 3).

**Table 1:** Average number of words and average percentage of first person pronouns per post caption, for the individuals and eNGOs, July 30-August 26, 2018.

		Individual Scientists					eNGOs			
		IND 1	IND2	IND3	IND4	IND AVG	ORG1	ORG2	ORG3	ORG AVG
**Average number of words per post caption**	**TRP**	30	26	36	27	**30**	33	24	28	**28**
	**IGP**	109	312	291	265	**244**	134	51	122	**102**
**Average percentage of captions that were first person pronoun words**	**TRP**	2.2	3.8	3.5	4.2	**3.4**	3.6	0.9	1.8	**2.1**
	**IGP**	5.5	5.1	5.0	5.0	**5.1**	1.3	1.3	2.0	**1.5**

### Interpersonal strategies

**Fig 4:**
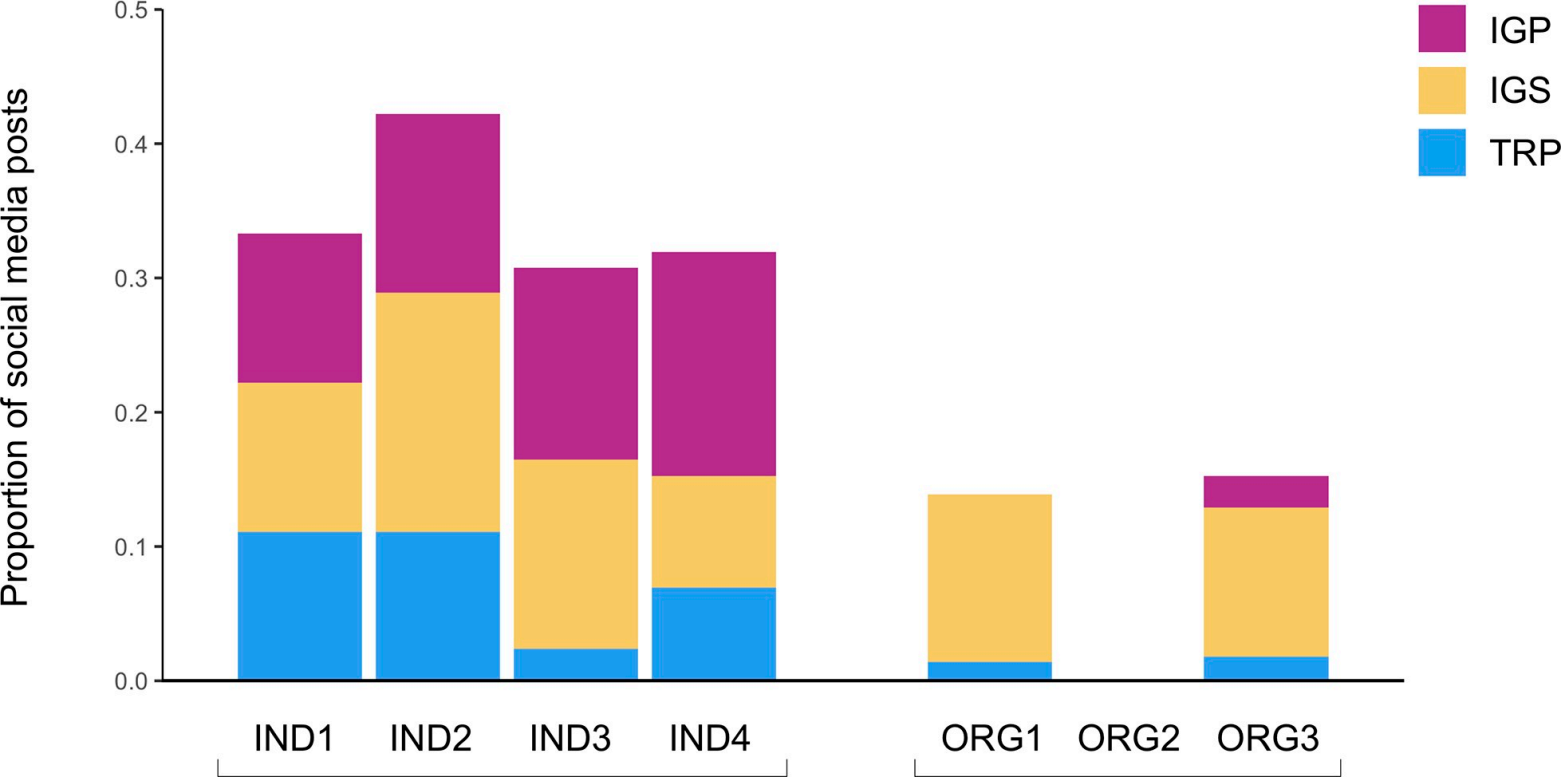
Proportion of social media posts by individuals and eNGOs containing selfies, July 30-August 26, 2018. Colours indicate relative proportion of posts with selfies in the TRPs, IGSs, and IGPs.

The seven communicators all noted in their interview responses that they aim to integrate interpersonal strategies into their social media activities. Some of these strategies are non-conversational, resulting in no direct interactions between the communicators and audience members. Six of the communicators stated that humanizing social media content is important for establishing personal connections with audiences. To humanize their organizations the representatives of ORG1 and ORG3 stated they display images of scientists or other staff members in posts. As one eNGO representative said, “It’s good for people to get to know who… the researchers or advocates are behind each of the stories and who’s working on them and why. I think [that’s] useful for people… that human aspect is important, and… giving people a chance to get to know who’s behind the controls is a good thing” (ORG1 Interview). However, the ORG1 and ORG3 representatives also stated that posting selfies and humanizing their organizations is one of their biggest social media challenges, particularly as the organization staff are often not willing to be seen in social media photos/videos, and because the organizations employ multiple staff members to create content for social media (ORG1 and ORG3 Interviews). In practice, ORG1 and ORG3 include selfies in a small fraction of their posts (14% and 15% of posts respectively), whereas ORG2 does not post any selfies on social media (Fig 4).

Selfies are a key means of humanizing the individual scientists since displaying their faces allows people to become comfortable with them. The individual scientists stated they use selfies to convey authenticity and to encourage/invite their audience to engage with them. As IND3 said, “I do try to be the most honest version of myself that I can display,” which “is important because it helps people to understand and also care about what you’re communicating” (IND3 interview). Similarly, IND2 noted: “that’s why I like to film in a selfie mode, because also it… puts a face on a scientist. People like to connect with other people” (IND2 interview). IND1 also expressed a similar view: “that’s one hundred percent to be human… even if you post a photo with your science, or with your code, or whatever… I think even in my facial expressions I try to make it about inviting people in” (IND1 interview). Selfie strategies are evident in the actual posting activity of the individual scientists, who collectively utilize selfies far more frequently than the eNGOs, incorporating selfies into more than 30% of posts (Fig 4). Additionally, selfie-style videos are important for the individuals, who noted they speak directly to their camera to convey a sense of talking directly to their audiences. The individuals believe these videos are especially effective for communicating on a personal level and establishing communicator-audience relationships. For example, IND3 explained how selfie-style videos feel very authentic and conversational:

I think video content, especially… a selfie-style video… feels pretty intimate actually. It feels like you’re having a one-on-one conversation, and it really helps… to build relationships with the audience. Because it feels very personal to have someone speaking right to you via the phone in your hand. (IND3 interview)

**Fig 5:**
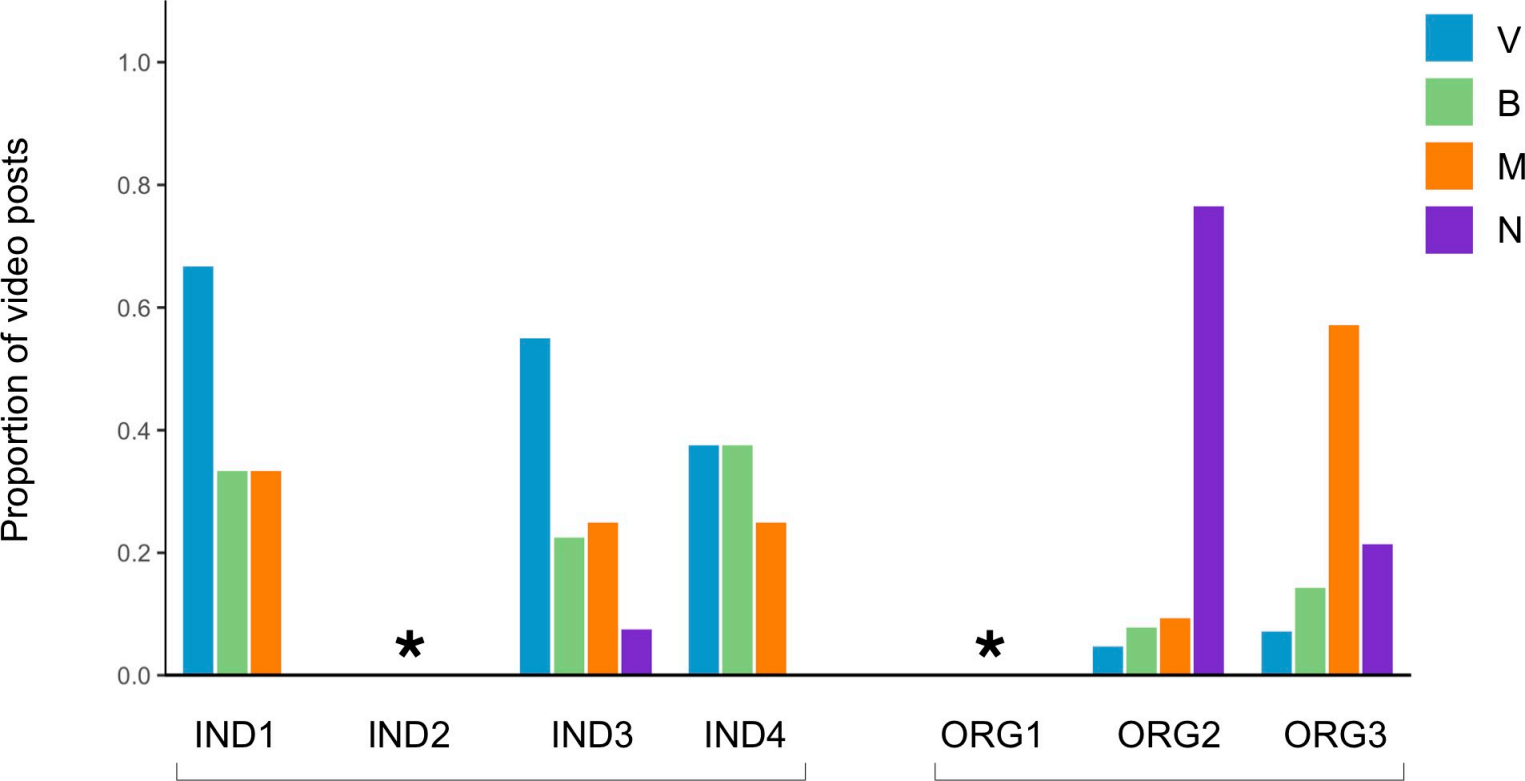
Proportion of video posts by individuals and eNGOs containing selfie-style audio (V), background audio (B), music (M), and no audio (N), July 30-August 26, 2018. *Two or no videos posted (IND2 and ORG1).

Selfie-style videos are commonly implemented as a strategy by the individual scientists, as a substantial proportion of their video posts (38–67%) include selfie-style audio (Fig 5). In contrast, the eNGO communicators rarely use selfie-style audio in their video posts (5–7%), generally opting for no audio at all, or music-based audio (Fig 5).

**Fig 6:**
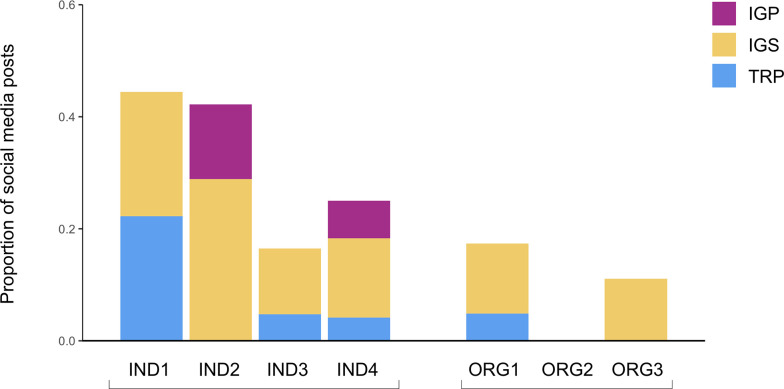
Proportion of off-topic posts by individuals and eNGOs, July 30-August 26, 2018. Colours indicate the relative proportion of off-topic posts in the TRPs, IGSs, and IGPs.

In addition to practices to humanize their social media activity, the communicators used non-conversational interpersonal communication strategies linked to the social media topics of their posts. Educating audiences through social media is an important goal of the eNGOs, and they give particular attention to the manner in which education is conducted. They emphasize a two-way model, rather than a top-down approach where information only flows from communicator to audience. For example, ORG1 pointed out: “I don’t know if it’s ‘teaching’… We don't want to be talk ‘down-y’” (ORG1 interview). The eNGOs also try to balance “heavier” educational/scientific content with “lighter” topics—such as posts focused on funny/interesting animals—and they use metaphors to make science content more accessible for their audiences. Similarly, the eNGOs stated they aim to make the content fun and interactive by presenting compelling information and mixing in humour. In addition, the eNGOs aim to build trust with their audiences by ensuring all of their posts are backed by scientific evidence. Overall, the social media activity shows that the eNGO communicators post consistently on topic (only an average of 9% of eNGO posts were off-topic, i.e., not clearly linked to the organization’s goals or mission, Fig 6), deciding to include entertainment and humour in posts topically linked to the organization’s goals/mission.

Similar to the eNGOs, the individual scientists exercise two-way communication practices to avoid talking down to their audiences and to balance the educational component of their social media activity with lighter content such as humour and entertainment. One individual emphasized this sentiment, describing the educational component as “teaching, but with an engagement model… helping people to engage with educational content” (IND3 interview). However, in contrast to the eNGOs, the individual scientists mainly balance the content by including personal social media topics—such as daily activities that might be unrelated to science—and expressed a clear intention to post personal content using IGSs. For example, IND1 discussed how posting personal content on IGSs helps to portray scientists as people, i.e., regular individuals who have interests outside of science:

I think that Instagram stories humanize [science] more than anything else. Just because they’re quick, they don’t have to be high quality… Sometimes [content is] not exciting enough to warrant a whole post on Instagram, but you know, people like seeing it on the stories. Because it’s a way for them to check in with me, and like, what I am doing between posts. (IND1 interview)

The individuals also focus on expressing emotions in their post topics, and try to authentically display themselves, and scientists more generally, as warm, kind people as opposed to strictly knowledge experts absent of approachable qualities. In addition to ensuring their posts are all evidence-based (a strategy emphasized by the eNGOs as well), the individual scientists work to establish personal connections with their audiences in order to build trust. In highlighting use of selfie-style videos, IND3 said, “Recording an off the cuff video just kind of… confers some level of honesty. Because it’s you just free stream talking as if in conversation. And so, I try not to overly produce anything. Because I want people to see… we’re just talking, this is not so serious. We’re just having conversations, let’s delve in” (IND3 interview). The social media data demonstrate that the individual scientists share a larger proportion of off-topic posts than eNGOs (an average of 32% of posts were off-topic), many of which are about everyday activities (Fig 6). The text analysis of social media posts via LIWC shows that individual scientists also use more first person personal pronouns in their posts than the eNGO communicators; 3.4% and 5.1% of words in captions posted by the individuals on Twitter and Instagram respectively were first person pronouns (Table 1). In comparison, the eNGOs used such pronouns less frequently (2.1% of words on Twitter, 1.5% of words on Instagram).

**Fig 7:**
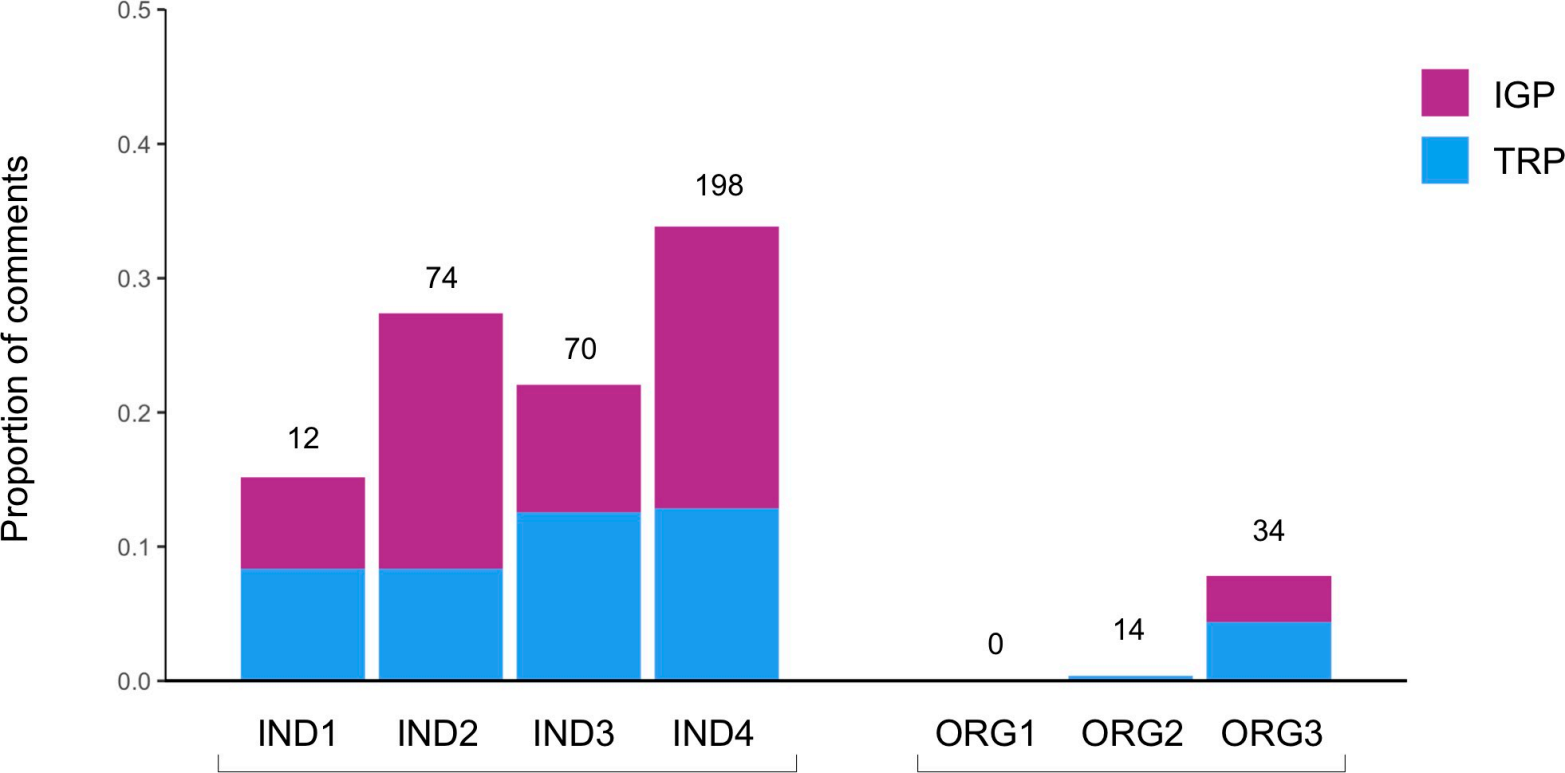
Average proportion of comments responded to per social media post by individuals and eNGOs, July 30-August 26, 2018. Colours indicate the relative proportion of comments responded to on TRPs and IGPs. Numbers on top of bars indicate the total number of comments responded to during the study period.

The seven communicators also implement interpersonal communication strategies via two-way conversations with their audiences. The eNGO communicators stated that they prioritize responding to audience comments on their posts, especially when people ask questions. The eNGOs also put calls to action (such as requests for audience members to sign petitions or join meetings) and/or questions in their posts, and endeavour to make their posts captivating, all designed to encourage audience members to participate in social media conversations. In addition, the eNGO communicators view two-way conversations as an opportunity to establish personal connections with their audiences and form communicator-audience relationships. For example, ORG2 said that “it’s difficult to build that relationship without having a conversation. So… enabling the opportunity to interact one-on-one with the individual… [is an occasion] to be able … to take that next step in that relationship” (ORG2 interview). Nonetheless, the eNGO communicators did not particularly feel they have been successful in forming communicator-audience relationships, as noted by ORG1: “I don’t feel like I have much of a personal relationship with the followers, no” (ORG1 interview). While the eNGO representatives stated that engaging with audience members was important, in practice, ORG1 and ORG2 respond to few, if any comments (responding to less than 1% of comments per post) (Fig 7). Although ORG3 responds to about 8% of comments per post, it still does so much less frequently than all individuals (who responded to an average of 15–34% of comments per post).

In the interviews, the four individual scientists also discussed interpersonal communication strategies via two-way conversations with their audiences. They prioritize responding to audience comments (particularly questions), put calls to action and/or questions in their social media posts to encourage a conversations, and strive to establish personal connections with their audiences and form communicator-audience relationships via two-way conversations. This view was obvious in a statement by IND3: “A lot of the time we’re just building relationships, we’re laughing. I’ll post something funny, and someone will reply… Further, it’s important for me to let people know that scientists do care about them… We care about individuals more than people realize… So it’s important for me to address people’s concerns, and talk with them, and share with them information that they’re curious about” (IND3 interview). In practice, the individual scientists respond to a substantially larger proportion of audience comments than the eNGOs (15–34% of comments per post (Fig 7)). The individual scientists also highlighted that they have been able to form communicator-audience relationships through their social media activity, as evidenced by a comment by IND4: “Yeah, [meeting up with an audience member in person for the first time] was great. It was weird in the fact that it wasn’t a complete stranger. So although it was the first time that you met them, you were talking to them like you had known them for ages” (IND4 interview). One individual scientist noted that although typical conversations on posts might be short, the conversations can extend beyond single posts once communicator-audience relationships are formed:

Oh my gosh, they’re ongoing. They’re very ongoing. There are many examples of people messaging me to ask for advice … and [they] almost always follow up. So I had one woman applying to a … program, and we actually even met in person because she happened to be visiting, and we exchanged some advice and conversation. And a year later she followed up and let me know she got into the program … and we had been chatting in the interim, but not so much. But many times people will follow up and let me know how it went, and say thank you, and say, “Oh I also learned this, you can tell people that next time” … So now we’ve turned a one-time interaction into a long-term resource, which I think is cool. (IND3 interview)

In contrast, the eNGO communicators noted during interviews their intention to build relationships with audience members through social media, but did not indicate that they had been successful in doing so.

### Audience engagement on communicator posts

Triangulation of the social media and survey data was carried out to understand why audience members decided to engage with social media posts shared by the communicators. The individual scientists receive more conversational engagement than the eNGOs, that is, the individuals receive more and longer comments, and generate a larger number of direct interactions with unique conversationalists (Table 2). The individuals receive 20–42 comments/post/10,000 followers on Instagram, and 0.8–60 comments/post/10,000 followers on Twitter whereas the organizations receive 1–4 comments/post/10,000 followers and almost no (0.05–0.4) comments/post/10,000 followers on Instagram and Twitter, respectively (Table 2).

**Table 2:** Average number of comments per post, number of words per comment, number of unique conversationalists engaged, percentage of conversationalists who interacted directly with the communicator, and percentage of survey participants who identified as scientists on social media, for the individuals and eNGOs, July 30-August 26, 2018.

		Individual Scientists					eNGOs			
		IND 1	IND2	IND3	IND4	IND AVG	ORG1	ORG2	ORG3	ORG AVG
**Average number of comments per post per 10,000 followers**	**TRP**	60	0.8	2	4	**17**	0.05	0.2	0.4	**0.2**
	**IGP**	25	42	20	41	**32**	3	1	4	**3**
**Average number of words per comment**	**TRP**	26	9	16	16	**17**	2	7	15	**8**
	**IGP**	19	11	24	26	**20**	5	5	7	**6**
**Number of unique conversationalists per 10,000 followers**	**TRP**	34	2	7	30	**18**	2	5	5	**4**
	**IGP**	16	69	24	108	**54**	30	27	14	**24**
**% of unique conversationalists who interacted directly with the communicator**	**TRP**	6	100	100	91	**74**	50	8	32	**30**
	**IGP**	65	97	78	99	**85**	17	0.06	52	**23**
**% of survey participants that self-identify as scientists on social media**	**TRP**	44	100	60	90	**73**	None invited	22	None invited	**-**
	**IGP**	57	43	42	67	**52**	0	0.6	None invited	**0.3**

A two-way conversation was defined as a comment that received at least one response, with both the commenter and respondent invited to complete the survey.

Comments on the individual scientists’ posts ranged from 11–26 words in length on Instagram and 9–26 words on Twitter (Table 2). In contrast, comments on the eNGO communicators’ posts ranged from 5–7 words on Instagram and 2–15 words on Twitter (Table 2). Although the total number of unique conversationalists varied across the two groups and platforms (Table 2), an average of 74% and 85% of unique users interacted directly with the individual communicators on their Twitter and Instagram posts, respectively (although IND1 on Twitter was far lower than the other individuals). An average of 30% of unique conversationalists interacted directly with the eNGO communicators on their Twitter posts, and an average of 23% did so on Instagram posts (Table 2).

Although direct message data were not collected (this information is not public in either Twitter or Instagram), all of the communicators indicated during the interviews that direct message engagement does not occur more frequently than comment engagement. Furthermore, although the eNGO communicators engage a majority non-scientific audience (0–22% of conversationalists across Instagram and Twitter were identified as scientific users), the individual scientists reach a mixed audience consisting of both scientific and non-scientific users–particularly on Instagram–with 42–67% of conversationalists identified as scientific users on Instagram, and 44–100% identified as scientific users on Twitter (Table 2). While mixed, scientists constitute a large proportion of the audience of the individual communicators.

The survey of conversationalists yielded a response rate of 10% (45 out of 425 invited to complete the survey). Most of the survey respondents were engaged on posts of the individual scientists (five on Twitter and 33 on Instagram), and seven were engaged on posts of the eNGO communicators (all from Instagram). The majority (62%) of respondents who identified their age were between 19–33 years old, with a smaller proportion (16%) aged 5–18 and 34–49 combined (Table 3). Only two of the survey participants were 50 or above. Most of the survey respondents who revealed their gender identified as female (82%) (Table 3). The respondents were also highly educated and science-associated overall: 83% of respondents had some level of post-secondary education, and 80% consider themselves part of the scientific community (Table 3). Although the majority of survey participants were well educated and science-associated, many users who participated in conversations on the posts of the science communicators were not scientists, especially those engaged with eNGO posts (Table 2).

**Table 3:** Participant age, gender, level of education, and scientific community association (“n” = the number who responded to each demographic question).

Age (n = 37)	Number of Participants
5–18	6
19–33	23
34–49	6
50–64	1
65+	1
**Gender (n = 34)**	
Female	28
Male	6
**Level of Education (n = 36)**	
Grade school or high school	6
Post-secondary and above	30
**Member of the Scientific Community (n = 45)**	
Yes	36
No	9

Some survey participants provided open text responses that explained why they engage in conversations on posts of the communicators, frequently expressing personal sentiments (emotional connections to the communicator and/or their posts) in their responses, rather than focusing on education or links to science. Those who prefer to engage in conversations on Twitter do so due to its short message length and focus on news/relevant information (Table 4). The participants who expressed a preference for Instagram drew attention to its visual nature, its communication features, and its ease of use/functionality (Table 4). Regardless of platform preference, the most cited reasons for using Twitter related to the participants’ work and their seeking news/information. In contrast, the participants use Instagram because of the platform’s visual nature, and for personal reasons such as self-expression, relationship-building, and connecting with friends/family (Table 5). Personal sentiments also emerged when the respondents wrote about their motivation for following particular accounts. Although they follow the communicators to receive new information, many also do so because they find the communicators (or the content) to be relatable (Table 5).

**Table 4:** Participants’ most preferred platform (n = the number who stated their most preferred platform).

**Reasons Participants Prefer Twitter (n = 7)**	**Code Frequency**
Relevant news/information	3
*“I use Twitter for advertising my research as well as keeping up-to-date with others’ research and upcoming research/news in my field*.*”*	
**Reasons Participants Prefer Instagram (n = 27)**	
Communication and outreach	10
*“Instagram is most frequently used as it allows easy and efficient communication with people*, *businesses and organizations*.*”*	
Visual content	9
*“I love that images are first and foremost with the caption then complementing this*. *This way of communicating speaks to me*.*”*	
Functionality/ease of use	5
*“I like Instagram because it has a really clear and easy to handle design and because it is really intuitive to use … I also really appreciate the experimental stuff Instagram does*, *like adding Instagram Stories*.*”*	

The quotations are from the survey responses.

**Table 5:** Participants’ reasons for using Twitter, Instagram, and for following/responding to the communicators (n = number of people who provided an open text response).

**Reasons Participants Use Twitter (n = 15)**	**Number of Participants**
News/information	8
*“I use Twitter to get news”*	
Work	3
**Reasons Participants Use Instagram (n = 26)**	
Personal	9
*“Instagram helps me express myself*.*”*	
Multimedia information (text and visual)	5
*“Instagram is photo and short caption/story based*.*”*	
**Reasons Participants Follow the Communicators (n = 26)**	
Relatability	10
*“It is interesting to read [about a] person who is going through the same [things] in life*.*”*	
Learn new information	8
*“They have engaging content that I enjoy*, *and I constantly learn new things from them about grad school*. *The insight is really interesting and important to me”*	
**Reasons Participants Respond to the Communicators (n = 19)**	
Personal connection	5
*“I just feel that I engage better with a single person rather than an organization*.*”*	
Know the person behind the account	4
*“[I] prefer to know the person I’m engaging with*.*”*	
Communicator is authentic	3
*“An individual is more likely to respond authentically*.*”*	

The quotations are from the survey responses.

A theme that emerged from all survey responses was the participants’ sense of personal connection with the communicators, which encouraged participation in conversations, particularly on Instagram, which the participants viewed as a more personal social media platform compared to Twitter. For example, one participant stated: “it seems personal and engaging (photos and captions) but without the threat of things getting out of hand or out of context like on Twitter.” The survey respondents also noted that Instagram is quite conducive to communication, illustrated by the participant who stated: “I’m most active on Instagram and it’s easy to make and respond to comments, posts, and stories.” When the respondents commented about their decisions to engage with the communicators, many (12 out of 19) did so in terms of personal connections, perceived authenticity of the communicator, and feeling that they knew the owner of the account (Table 5). For example, one wrote, “for me it is easier to contact a person instead of an organization with 'unknown faces' behind it.” Another respondent described a sense of comfort in interacting with organizations that are comprised of known individuals, “I use social media for work so I know there are ‘individuals’ behind the organization… However if I didn’t know the organisation, then I would be less likely to reply.”

When queried about establishing relationships with the communicators, 24 respondents added explanations, and 13—both those that do and do not feel that they have formed relationships with the communicator—commented specifically about two-way conversations. One did not feel an opportunity to form a relationship was presented, because direct interactions had only occurred with other users, not the communicator: “I don't think [the communicator has] ever responded to anything I've said on their post, responded to one of my posts, or anything of the like. It's impossible to feel any link if it's not reciprocal.” In contrast, those who formed relationships emphasized the dialogic interactions: “we have commented back and forth to each other as well as [direct messaged] in the past!” Two others expressed similar comments: “we talk in private as well as I do with my friends”; and: “I often message [them] if I need to know anything about being in academia, because I am new to it and [they are] really helpful.” One respondent also stressed that the way posts are presented on social media is crucial, and can result in a relationship-type connection in the absence of direct interactions with the communicator:

We don’t talk, but their welcoming demeanor and friendliness makes learning science personal. It feels like engaging with a friend. Their method of communication makes science a more fun and accessible conversation. You feel like you are involved, and you can always put forth your input without judgement—something that is super important because science can appear condescending to a lot of people. It’s constant learning and that’s all that matters.

## Discussion

Recognizing that social media provide a means of two-way interactions—which research suggests are crucial for effective communication [33, 34]—individual scientists and NGOs are increasingly using social media platforms to communicate with their audiences and promote science literacy [46, 47, 68, 75, 106]. However, individual and NGO communicators have had difficulty fostering two-way exchanges with their audiences on social media [33, 106]. With evidence that the way in which communicators use social media plays an important role in determining audience engagement [e.g., 31], this study investigated how individual and NGO communicators approach sharing scientific information on social media, and the strategies they apply to engage with their audiences (RQ1).

The individual and eNGO communicators in this study implement three strategy “filters” in a hierarchical manner to guide their posting activity. First, both communicator groups follow implicit platform conventions when sharing posts on social media. All of the communicators follow a similar approach to ensure their posts are consistent with audience expectations, for example, focusing on more news-centric content in Twitter posts (TRPs), and more visually interesting content in Instagram posts (IGPs).

Second, both of the communicator groups are intentional in how often they post on the social media platforms, as well as in the types of media they use in posts. This activity “filter” is applied differently between the communicator groups. For example, the eNGOs implement a more scheduled approach, typically posting frequently, at regular intervals, and mainly on Twitter. In contrast, the individual communicators are more flexible in how often they post, and share information mainly via Instagram, particularly Instagram stories (IGSs). However, the activity strategies applied by the communicators do not link directly with conversational engagement on their social media posts. When comparing proportional engagement between the communicators (normalizing engagement to the number of followers for each communicator), ORG2—which posts far more frequently than the other communicators—receives fewer comments than the other communicators, and is in conversations with fewer unique users. IND1 and IND2 post less frequently than the other communicators, but they do not receive lower engagement with regard to user comments or unique conversationalists. A link between media type used (frequency of text, images, videos) and conversational engagement is also not obvious. Furthermore, a connection between the platform given priority in practice (i.e., the platform posted to most frequently) and conversational engagement is not evident, as all of the eNGOs receive more engagement on IGPs than TRPs despite posting more frequently on Twitter than Instagram.

The data in this study show that the implementation of interpersonal social media strategies by the communicators (i.e., the third strategy “filter”) encourages conversational engagement (RQ2). The next section discusses the characteristics of interpersonal strategies that encourage communicators and audiences to participate in two-way conversations (RQ2).

### Interpersonal communication strategies and social media engagement

A variety of interpersonal communication strategies have been demonstrated to affect social media engagement [62], many of which are used by both the individual and eNGO communicators. For example, both the individuals and eNGOs actively invite people to participate in conversations on their posts, which is important because this approach encourages engagement, an opportunity that would otherwise be missed [25, 62, 107]. However, the individual scientists more comprehensively implement interpersonal communication strategies. First, the individuals post selfies and selfie-style videos more frequently than the eNGOs. This difference is noteworthy for engagement, as social media users are more willing to comment on posts by communicators whom they know, and more likely to initiate conversations with communicators who are familiar to them [26, 29, 69]. Furthermore, previous research shows that speaking directly to social media audiences through the camera—as is common practice for the individuals in selfie-style videos—can personally connect communicators with audience members and help to build trust and establish communicator-audience relationships, even in the absence of direct communicator-user interactions [27, 84, 108, 109]. In addition, research on interpersonal communication has shown that this form of communication entails establishing relationships among the participants [105]. The results of this study support the link between selfie-style posts, two-way conversations, and communicator-audience relationships, as the individual scientists receive more engagement than eNGOs overall, and successfully formed relationships with their audiences, even in the absence of direct interaction (as corroborated by the survey responses). The frequent use of selfie-style image and video posts appears to be an effective strategy to build trust, establish communicator-audience relationships, and stimulate discussions of science on social media, which science communicators could implement to encourage effective science communication.

The expression of interpersonal sentiments in posts is also important for social media engagement, as recent research suggests that content characteristics affect engagement. For example, when users see social media posts similar in nature to their own, they are better able to connect with the content on a personal level and engage with it [28, 30]. Although both communicator groups discussed strategies to make their social media content more relatable, the individual scientists receive more engagement in terms of two-way conversations than eNGOs overall, which may be because the former choose to focus on posting personally-relatable content. When the individual scientists post off-topic content such as day-to-day activities and frequently use first person pronouns in posts, they create relatable, shared stories that are thought to be key for audience engagement [26, 110]. In fact, posts with a personal sentiment or message (including those without any science content) can surpass scientific posts in terms of engagement, even on science-focused accounts [107]. A link between engagement and personal content was evident in the survey responses, which showed users choose to follow communicators with whom they can relate. The results of this study suggest that the use of personal and relatable social media content promotes more two-way interactions in social media with science communicators than would otherwise occur.

Previous studies show that using two-way conversations to form communicator-audience relationships is important for social media engagement. Two-way conversations can result in personal connections between users and organizations, and cultivate positive organization-public relationships, which are crucial because organizations often have difficulty in retaining engaged users on social media [62, 111–113]. However, the means through which relationships are formed between organizations and users on social media goes beyond direct interactions, as research shows that a significant number of users are influenced by the interactions they see online. When communicators engage with an individual, they are indirectly affecting relationship perceptions for others who observe the interaction, even when no direct communication takes place with the latter [114]. Additionally, the survey responses demonstrate that communicators are capable of establishing relationships with audience members through the use of personal sentiments even in the absence of direct interactions. Therefore, because the eNGOs currently respond to a smaller proportion of audience comments compared with the individual scientists, the eNGOs engage in fewer two-way conversations and therefore may be more limited in their ability to form communicator-audience relationships than individuals. This outcome is supported by this study: two-way conversations between individual communicators and audience members resulted in the establishment of communicator-audience relationships, whereas the eNGOs communicators were less successful in forming relationships with their audiences. Furthermore, because more conversations can result when communicators form relationships with their audiences (as discussed above), two-way conversations and communicator-audience relationships appear to be mutually reinforcing. Consequently, focusing on responding to audience comments to form communicator-audience relationships is likely an effective strategy to create sustained social media engagement between science communicators and their audiences. One of the individual scientists emphasized that conversations are not limited to individual posts; instead, when communicators establish relationships with their audiences, the relationships allow conversations to extend beyond a discrete instance, and into a larger, ongoing conversation. Therefore, science communicators will benefit by being responsive to social media comments and working to establish communicator-audience relationships in order to facilitate longer-term, ongoing conversations about science [115].

### Non-scientific audience engagement

Both the individuals and the eNGOs stated that they specifically target non-scientific audiences with their social media activity (although the communicators do not limit their audiences to non-scientific users alone). In the interviews, all seven communicators pointed out that they generally use Instagram to reach non-scientific audiences, as they feel the platform attracts a larger population of non-scientific users than Twitter. Studies have shown, however, that the educational distribution of users on Twitter and Instagram is relatively similar [116, 117]. The apparent mismatch between the perception of the communicators and subscriber base of the two platforms may be due to the topics of focus by the communicators on social media and the audiences that they have built. To date, scientists have typically been heavier users of Twitter than Instagram, and because the communicators post an abundance of science-based content [78, 79], they may attract more scientists via Twitter than Instagram. Furthermore, education level does not necessarily equate to science literacy. In this study, all of the communicators except IND1 appear to engage a larger proportion of scientific users in conversations on Twitter than on Instagram. Moreover, a higher proportion of users in conversations on posts by the eNGOs are non-scientific compared to the individual scientist communicators. This result is likely a consequence of the differences in target audiences, topics, and social media goals among the communicators indicated during interviews. Nonetheless, the individual scientists engage a mixed (scientific and non-scientific) audience on social media, particularly on Instagram. Therefore, as this study shows, focusing on Instagram as a platform to reach non-scientific audiences for science conversations could be an important science communication strategy.

### Interpersonal communication afforded through Instagram

Determining the extent to which Instagram fosters social media engagement is another informative outcome in this study. Not only did a greater number of two-way conversations take place on Instagram than Twitter for nearly all of the communicators (including the eNGOs that do not prioritize the platform in practice), Instagram was favoured by the communicators and survey participants for conversation-related uses overall, particularly illustrated by their understanding of accepted social media practices. The visual, informal, multi-functional, cordial, and multimedia-focused nature of Instagram (both posts and stories) contributes to it being a more conversational platform than Twitter. Science communicators could capitalize on this functionality of Instagram to encourage more conversations and informative two-way science communication with diverse audiences.

### Implications

**Fig 8:**
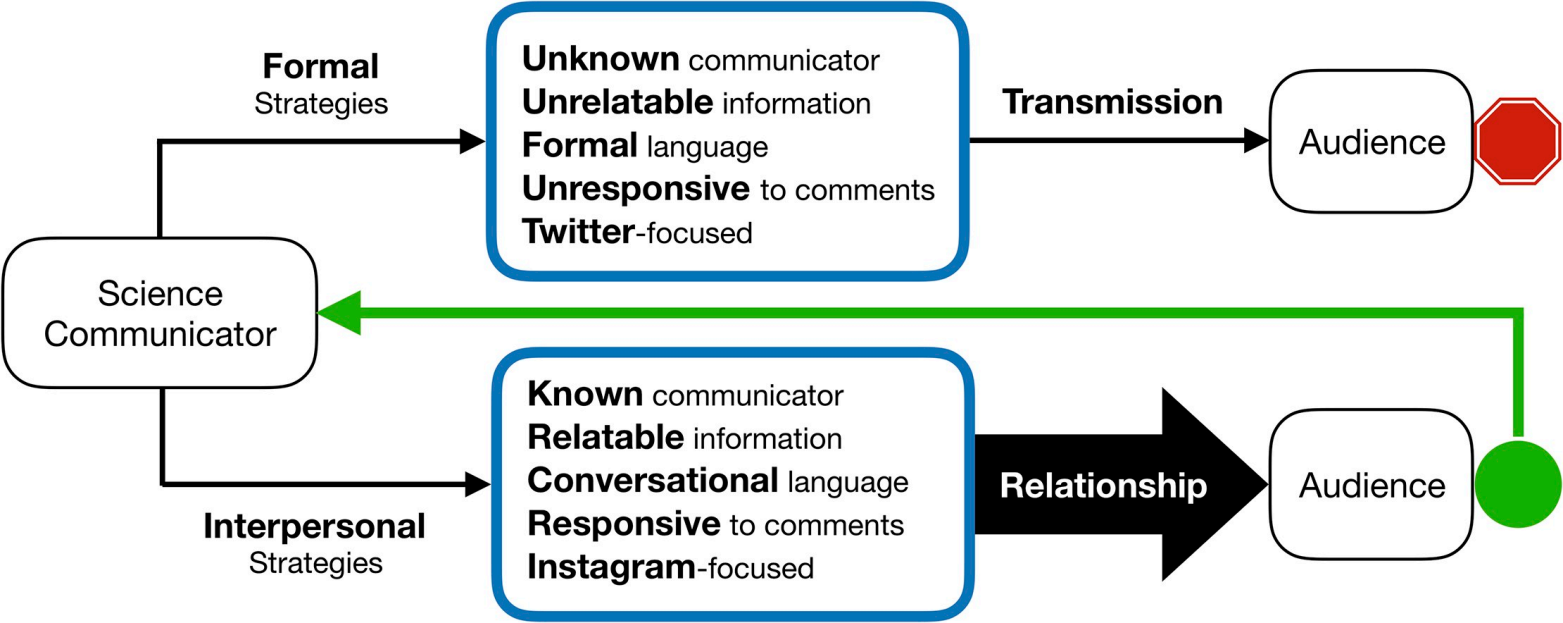
Representation of formal vs. interpersonal communication strategies on social media. Formal strategies are not sufficient to establish a relationship between audience and communicator, resulting solely in a transmission pathway. Interpersonal strategies act as enablers to information flow, resulting in communicator-audience relationships, which promote two-way conversations sustained over time.

This study is especially informative for understanding characteristics of science communication on social media, and could contribute to dialogic theory on science communication more broadly, as the results highlight factors that play an important role in fostering two-way exchanges [62, 106, 118]. The use of more formal methods typical of traditional science communication practices, i.e., through transfer of publications (data and information in various forms, e.g., peer-reviewed research papers) [119–122], often results in a transmission pathway, where conversations are limited between communicators and their audiences (Fig 8). In contrast, the implementation of interpersonal strategies by science communicators promotes the formation of communicator-audience relationships and encourages audiences to participate in more two-way conversations, resulting in positive feedback effect (Fig 8). Crucially, because the interpersonal communication practices observed in this study mainly relate to *how* content is shared rather than *what* information is shared or *who* it is shared with, such strategies are applicable to a wide diversity of subjects and audiences. Therefore, science communicators of all types (individual scientists, organizations, government agencies, etc.) can communicate interpersonally with citizens about a variety of scientific topics for which research information is relevant to make policy decisions, promoting citizens to be more scientifically engaged in environmental, health, and other issues.

For organizations such as eNGOs that are communicating with large non-scientific audiences, the potential to engage citizens in the science of environmental issues through interpersonal strategies is high. Importantly, because organizations do not operate in the same manner as individual scientists, they may be more limited in their ability to adopt interpersonal communications (for example, organizations are staffed by multiple individuals, and/or may be hesitant to share off-topic content or use first person pronouns due to organization culture) [123, 124]. Furthermore, organizations face particular challenges and risks when using social media, such as losing control of the narrative of messages or being portrayed as less authoritative, which are not eliminated with the implementation of interpersonal strategies. In such cases, organizations could develop specific guidelines for implementing interpersonal communication into their social media activities in a manner consistent with higher-level organization practices. Nonetheless, because the eNGOs in this study share many goals with the individual scientists (such as encouraging two-way science conversations), eNGOs could apply interpersonal communication strategies—through a “spokesperson,” for example—and promote improved scientific literacy in their audiences on environmental issues that the organizations are engaged with.

Although this research investigated science communication on social media, the interpersonal strategies observed to promote conversations with citizens are applicable to all science communicators in diverse environments. Science communicators working to engage their audiences with environmental research information can apply interpersonal techniques offline as well as online. For example, communicators could utilize interpersonal communication strategies to establish relationships with relevant stakeholder groups involved in participatory policy processes and gain a better understanding of stakeholder concerns, ultimately leading to greater cooperation and more effective management decisions that are inclusive of stakeholder values [115].

### Limitations and future work

The sample size of communicator participants was selected to examine the research question in a detailed and qualitatively data-rich manner rather than be representative of all scientists and eNGOs communicating on social media; nonetheless, increasing the number of communicator participants could reveal whether the conclusions of this study hold across a broader group of communicators and their audiences. Additionally, a longer period of study than was the source of data in this research, would provide further insights into communication patterns, such as how social media behaviours may be changing over time, regarding platform functionality and the way in which users employ social media tools (for example, a new feature called Instagram TV was instituted while this research was in progress). The ways in which social media research is conducted may also be required to change over time as the relationship between researchers and platform providers evolves and data access shifts [125, 126]. The study was focused on Twitter and Instagram; future work could include other popular social media platforms such as Facebook and YouTube to advance understanding of the effects of interpersonal communication on engagement across more platforms. The communicator participants in this study share slightly different information on social media (i.e., the individual scientists focused mainly on a range of science topics, whereas the eNGOs included more politics and advocacy, with science aspects), which could affect audience engagement. Further research could compare individual scientists and eNGOs focusing on a single science topic to identify any effect of content topic on audience engagement.

The demographic concentration of the survey participants tended toward younger, highly educated respondents. Future work could use sampling techniques to evaluate whether links exist between demographic characteristics and the choice to participate in social media conversations, as well as survey a larger number of audience members to draw broader representative conclusions. Furthermore, conversation quality and message framing were not measured to determine the extent to which social media conversations were scientifically meaningful and learning-oriented, or how messages were framed. Additional investigation into social media as tools to facilitate a participatory model of communication could advance understanding of conversation quality. Evidence from the survey in this study suggests that communicators are positively influencing audience behaviour. For example, 44% of the survey participants (n = 41) feel inspired by communicator posts to make behaviour changes in regard to the natural environment. Therefore, future research that focused on conversation quality could provide additional insight into the effectiveness of science communication to influence behavior. Determining deeper understanding of the extent to which communicators are reaching non-scientific audiences, and how communicator-audience networks are structured and operate, could be obtained through studies that investigate how to measure the level of effectiveness of conversations in communicator/audience interactions, the role of communicator/audience networks, and the presence of lurkers in such networks.

## Conclusions

A social media presence by itself is not sufficient for successful communication; how social media tools are used to encourage two-way conversations is an important determinant of engagement [25, 118]. Both the individual and eNGO communicator groups in this study share similar communication goals and conveyed strong awareness of strategies known to be effective for science communication (such as two-way conversations). The two communicator groups apply interpersonal communication strategies differently in their social media activity. One difference that emerged is their overall application of interpersonal communication strategies. The individual scientists particularly focus on making themselves known and relatable communicators throughout their social media activity, and on establishing relationships with their audiences. In practice, the individuals achieve this outcome by posting more selfies (images and videos), posting more off-topic content, responding to more comments, and using more personal pronoun-prominent language than the eNGOs achieved. The individual scientists also prioritize Instagram over Twitter (and particularly Instagram stories), which more readily supports the implementation of interpersonal communication strategies than Twitter. This emphasis by the individual scientists on interpersonal communication promotes the formation of communicator-audience relationships, encouraging more two-way conversations and generating greater numbers of opportunities to form relationships with their audiences than the eNGOs. In other words, the results of this study show that a combination of interpersonal communication strategies, and their application throughout the social media activity of science communicators via the features of the social media platforms, especially in Instagram, play an important role in determining audience participation in two-way conversations, and ultimately affect how audience members engage with communicators over time.

## Supporting information

S1 Table Codes and definitions used to characterize Twitter post, Instagram post, and Instagram story content. (TIF)
