# Supplementary figures and images for: Using interpersonal communication strategies to encourage science conversations on social media
Source: PLoS One. 2020 Nov 10;15(11):e0241972. doi: 10.1371/journal.pone.0241972 (PMC7654796; doi:10.1371/journal.pone.0241972)

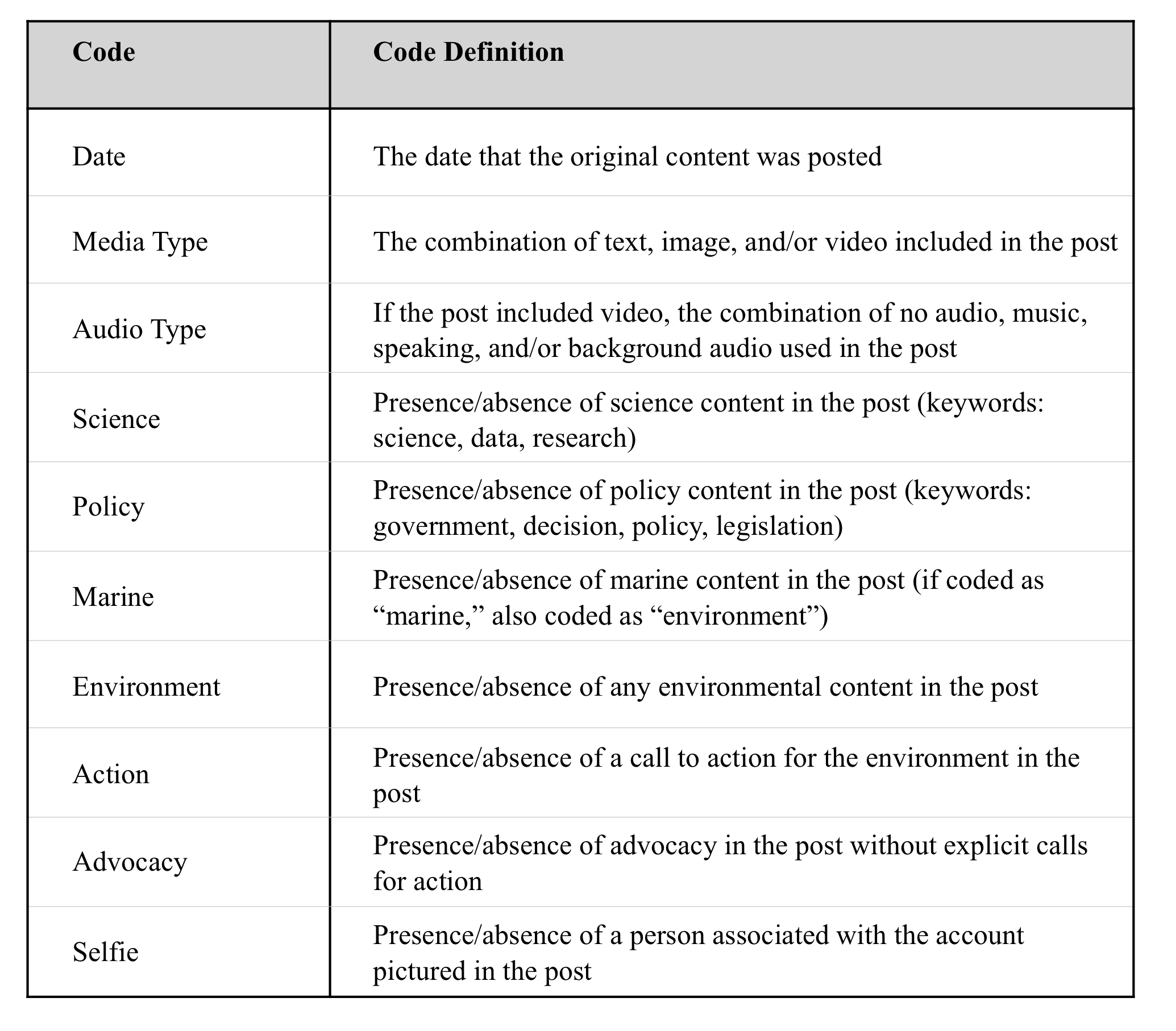

Supplement: S1 Table — (TIF) [file pone.0241972.s001.tif]
